# Casting wider than U-Nets: evaluating the role of novel machine learning applications in radiation therapy

**DOI:** 10.1093/bjrai/ubag018

**Published:** 2026-09-22

**Authors:** Osama Omrani, Sayan Biswas, Rajesh Jena

**Affiliations:** Department of Oncology, University of Cambridge, Cambridge, CB2 0AH, United Kingdom; Cambridge University Hospitals NHS Foundation Trust, Cambridge, CB2 0QQ, United Kingdom; Department of Mathematics, Jadavpur University, Kolkata, West Bengal, 700032, India; Department of Oncology, University of Cambridge, Cambridge, CB2 0AH, United Kingdom; Cambridge University Hospitals NHS Foundation Trust, Cambridge, CB2 0QQ, United Kingdom

**Keywords:** radiation oncology, artificial intelligence, deep learning, machine learning, image processing, precision healthcare

## Abstract

Radiation oncology has proven an early adopter of machine learning (ML) and deep learning (DL) owing to its image-centered workflow and data richness. DL auto-segmentation of normal structures is already guideline-endorsed, with self-configuring nnU-Net delivering near-state-of-the-art accuracy and widely adopted. In this review, we explore advances in ML algorithms in processing and interpreting imaging-based data beyond normal tissue auto-segmentation with applications upstream and downstream of this task, highlighting training data volume and best-in-class architectures. Applications discussed range from diagnostics with “virtual biopsies” and automated staging to radiotherapy planning with gross tumor volume and clinical target volume (CTV) prediction. We illustrate the developments of cutting-edge transformer-based designs and foundation models, but also the important role of lightweight, task-specific classical ML models. Data scarcity may be eased by developments in few-shot learning (FSL) and transfer learning strategies. We also consider how end-to-end automated CTV delineation may require an agentic approach to mimic the reasoning of a radiation oncologist, capable of processing multi-modal data with clinical context. Together, these advancements point toward a future where ML will become increasingly central to radiation oncology practice. However, implementation of these tools beyond normal tissue auto-segmentation remains limited and will require careful assessment and validation in the clinic.

## Introduction

Radiotherapy has been an early adopter of machine learning (ML) and artificial intelligence (AI) technologies, partly because many radiotherapy problems resemble core computer-vision tasks such as classification and segmentation. This dependence on image segmentation has driven the early clinical uptake of normal-tissue auto-segmentation.^1^ U-shaped network (U-Net), a deep learning (DL) encoder–decoder architecture introduced for biomedical image segmentation, and later self-configuring variants such as nnU-Net, now form the foundation of contemporary radiotherapy auto-contouring.^2^^,^^3^ However, broader applications of ML in radiation therapy are still mainly in the research domain. The clinician working in this space would benefit from understanding how ML is being used in these applications, where the potential benefits may lie, and the factors that limit their performance.

The radiation oncologist’s workflow and the patient cancer journey offer many opportunities to deploy ML. Radiology has led the experimentation and deployment of diagnostic ML, aided by readily available data and incredible potential clinical benefits.^4^ We will review the advances in imaging-based ML and multimodal integration, then consider applications in radiation oncology beyond U-Net normal-tissue auto-segmentation, highlighting clinical utility and promising methodologies in each case.

## Methods for processing imaging data

Diagnostic radiology has shown how much clinically useful information can be extracted from imaging data. ML models now support tasks such as lung-nodule detection, pulmonary embolism diagnosis, and, with large language models (LLMs), radiology-report generation.^5–7^ Given the rapid evolution of techniques for analyzing imaging data, we first provide an overview of the main approaches before turning to applications in radiation oncology (Figure 1).

**Figure 1:**
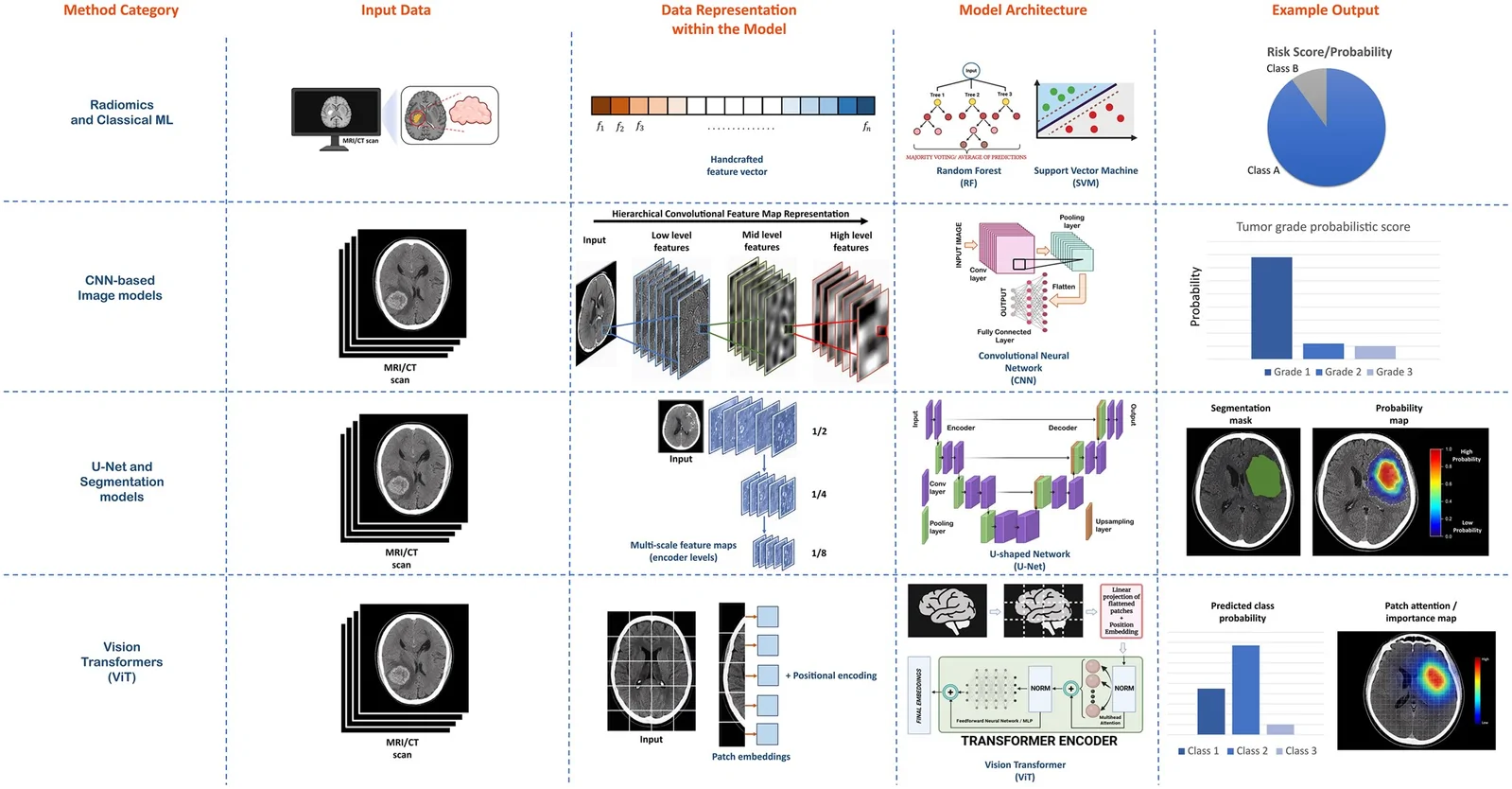
Representative machine-learning architectures for imaging-based applications in radiation oncology, illustrating differences in data representation, processing, and example output. Abbreviations: CNN, convolutional neural network; RF, random forests; SVM, support vector machine; U-Net, U-shaped network; ViT, vision transformer.

### Radiomics and classical ML models

Radiomics represents a specialized field within image analysis in which quantitative features are extracted from standard imaging modalities such as CT, MRI, and PET. Extracted from entire image sets or regions-of-interest, they capture first-order (intensity), second-order (textural), shape and higher-order features. Higher-order features apply mathematical transforms and filters to capture complex local and global spatial patterns.^8^

Radiomics relies heavily on classical ML algorithms, including random forests, support vector machines, k-nearest neighbors, naive Bayes, and ensemble methods, to map imaging features to clinically relevant outputs. Nevertheless, reproducibility and standardization remain major challenges in radiomics and are frequent criticisms of traditional radiomics studies. Variability in scanner acquisition, reconstruction protocols, segmentation, preprocessing, and software implementation can all influence extracted feature values and limit generalizability. To address this, the Image Biomarker Standardization Initiative has developed standardized radiomic feature definitions and processing recommendations, helping to improve cross-platform consistency and reproducibility. Recognition of these limitations is important when considering radiomics in conjunction with DL approaches, particularly for the development of robust, interpretable, and clinically translatable imaging biomarkers.^9–11^

### DL models

In addition to classical ML models, DL, a more advanced form of artificial neural networks (ANNs), has achieved significant success across medical imaging tasks including reconstruction, noise reduction, predictive modeling, clinical planning, and decision-support systems.^12^

DL learns patterns directly from raw data through multiple hidden layers, allowing progressively more abstract representations to emerge without handcrafted feature engineering. This ability to model complex, high-dimensional data has made DL central to modern medical imaging analysis.^13^

Convolutional neural networks (CNNs), inspired by human visual perception, have proven highly effective in medical image classification tasks. CNNs learn hierarchical feature representations directly from imaging data, significantly reducing reliance on handcrafted feature extraction processes traditionally required in radiological assessments. A notable advancement in CNN architectures is the Residual Network (ResNet), which addresses the challenge of training very deep networks by introducing residual, or skip, connections that facilitate gradient flow and improve learning efficiency.^14^^,^^15^

U-Net integrates CNN and encoder-decoder principles. Its U-shaped architecture utilizes a contracting path to extract hierarchical features and an expanding path to reconstruct spatial information and generate a segmentation map.^2^ U-Net has become the benchmark for medical image segmentation due to its accuracy in resolving fine structural details. Since then, there has been significant improvement in methods to improve feature selection, training efficiency, and segmentation accuracy.^3^ Here, we find the great strides taken in normal tissue segmentation, which has become ever more present in radiotherapy planning, with a wide range of commercial options available on the market.^16^^,^^17^

CNNs capture local structure well but are less suited to long-range relationships. Transformers address this through self-attention, allowing image patches to interact irrespective of geometric distance. In Vision Transformers (ViTs), images are represented as sequences of patches, conceptually analogous to tokens in language models. This can improve modeling of global anatomical context, although usually requires larger labeled datasets as the model starts with fewer built-in assumptions.^18^^,^^19^

Few-shot learning (FSL) addresses the challenge of generalizing from limited labeled data. Approaches include transfer learning and meta-learning strategies such as data augmentation, metric-based, model-based, and optimization-based methods. In medical imaging, FSL is attractive because data are often scarce, costly to acquire, and labor-intensive to annotate.^20^^,^^21^

## From algorithms to tools: applications within radiation oncology

### Virtual biopsy

Virtual biopsy has attracted major interest in oncology because imaging may be used to infer tissue characteristics, molecular classification, and genomic profiles without invasive sampling.^22^ Tissue biopsy remains essential, but it carries procedural risk, may be infeasible in some locations, and samples only 1 site at 1 timepoint.^23^ By contrast, virtual biopsy could characterize whole tumors longitudinally, capture heterogeneity, and support treatment selection, particularly in neuro-oncology where tissue acquisition can be high risk or impractical.^24–26^

Predictive models have been produced for the 5 main molecular markers of gliomas including isocitrate dehydrogenase (IDH) mutation, TERT-promoter mutation, 1p/19q co-deletion, MGMT-promoter methylation, and ATRX loss.^27–31^ As summarized in Table 1, pooled review-level performance is encouraging, with AUCs of about 0.85 for IDH, 0.87 for 1p/19q, 0.89 for TERT, and 0.81-0.87 across broader DL reviews of multiple markers. However, this literature is heterogeneous and dominated by retrospective single-center studies with limited external validation. Study quality is also inconsistent: in Di Salle et al.,^28^ the highest subgroup performance (AUC 0.92) came from studies with very low radiomics quality scores, raising concern that the best reported results may partly reflect methodological weakness rather than true generalizability. Similar concerns recur across reviews of 1p/19q, TERT, and multimarker DL studies.

**Table 1:** Summary of review articles addressing ML/DL approaches to virtual biopsy in neuro-oncology applications.

Review article	Clinical context	Population	Model algorithms	Validation strategy	Study quality assessment	Pooled sensitivity	Pooled specificity	Pooled AUC	Limitations
**Le Guillou Horn et al.^27^**	Scoping review of MRI-based radiogenomic prediction (MGMT, IDH, 1p/19q, EGFR, etc.) in gliomas/glioblastomas	17 Included primary studies (gliomas/glioblastomas), models trained with 2527 patients; study sample sizes 38-333	CNN, ResNet, DenseNet, U-Net, transfer-learning (AlexNet, GoogleNet, and InceptionV2)	Narrative synthesis (scoping review). Reports varied internal validation, external validation rare	NR	N/A	N/A	NR	Limited size and homogeneity of datasets; lack of well-defined external validation
**Di Salle et al.^28^**	Radiomics-based prediction of IDH mutation status in diffuse gliomas (MRI/PET/CT; GBM-grade 4 oriented inclusion)	26 Studies; 3280 patients	Radiomics feature extraction + classifiers (varies across studies); imaging modalities include MRI (±PET/CT)	Systematic review + bivariate meta-analysis; subgroup/meta-regression by RQS, grade, sequences/modalities; sensitivity analysis excluding potential overlap	QUADAS-2 + RQS; low RQS overall (mean 10.6 ± 3.3)	0.81 (95% CI 0.76-0.83)	0.80 (95% CI 0.76-0.83)	0.85	Globally low RQS; significant heterogeneity; small samples for subgroup analyses.
**Ahmadzadeh et al.^29^**	MRI-derived radiomics prediction of 1p/19q codeletion status in glioma	28 Studies qualitative; 10 studies in meta-analysis; study sample sizes 26-399	Mostly handcrafted radiomics + classifiers (RF most common); 2 studies used deep radiomics features; PyRadiomics common	Bivariate model meta-analysis; prioritized external validation, else HOIV, else training; subgroup and sensitivity analyses; publication bias via Deeks	QUADAS-2 + METRICS; METRICS: 5 low, 12 moderate, 11 good	0.82 (95% CI 0.67-0.91)	0.80 (95% CI 0.70-0.88)	0.87 (95% CI 0.84-0.90)	Meta-analysis relied on derived/approximated metrics due to missing contingency data; only 5 studies had external validation; potential small-study effects.
**Farahani et al.^30^**	MRI-based deep learning (DL-based radiomics) prediction of multiple glioma molecular markers	Glioma patients across 43 studies; 30 in meta-analysis; study sample sizes 42-985 patients	DL radiomics including CNNs, hybrid DL and radiomics, transformers/attention; segmentation often CNN/U-Net variants; Python frameworks (PyTorch/Keras/TensorFlow)	Bivariate random-effects meta-analysis when ≥5 studies; pooled estimates mainly reported for validation cohorts; meta-regression and subgroup analyses for heterogeneity	Modified QUADAS-2 + CLAIM + RQS; median RQS 16 (range 11-22/36); 4 studies high risk of bias	MGMT: 0.74 (95% CI 0.66-0.80); ATRX: 0.79 (95% CI 0.67-0.87); TERT: 0.81 (95% CI 0.72-0.87)	MGMT: 0.75 (95% CI 0.65-0.82); ATRX: 0.85 (95% CI 0.78-0.91); TERT: 0.70 (95% CI 0.61-0.77)	MGMT: 0.81; ATRX; 0.87; TERT: 0.81	Variability in study settings and data complicates clinical translation; moderate RQS; limited open code.
**Habibi et al.^31^**	MRI-based ML/DL prediction of TERT promoter mutation status in glioma	22 studies; total 5371 glioma patients; study sample sizes 16-129	Multiple ML/DL (eg, LR/LASSO/SVM/RF/XGBoost/CNN/ANN; mixed across studies)	Internal CV/holdout; 4 studies included external validation	CLAIM + QUADAS-2	0.86 (95% CI 0.78-0.92)	0.80 (95% CI 0.72-0.86)	0.89 (95% CI 0.86-0.91)	Limited number of studies in meta-analysis; mostly retrospective single-center small samples; heterogeneity; few external validations; manual segmentation variability

For systematic reviews and meta-analyses, quality assessment is also included.

Abbreviations: N/A, not applicable; NR, not reported; RQS, radiomics quality score; QUADAS-2, quality assessment of diagnostic accuracy studies 2.

Although these models have predominantly been CNN-based, recent evidence suggests that transformer-based networks are fast becoming an alternative option for noninvasive molecular classification. Niu et al^32^ combined 2 cross-scale ViTs (CrossFormer) with a radiomics model in an ensemble trained on 1185 multi-center cases. Each CrossFormer takes MRI slices with the largest tumor cross-section plus neighboring slices from an MRI sequence (T1CE or T2 FLAIR) and splits the feature map into both small and large patches, communicating with each other at all scales, with the network learning both fine margins and overall shape in one pass. Their 2.5-D fusion model sustained external validation AUCs of 0.855-0.904 for the clinically aggressive IDH-wild-type/TERT-mutant genotype and delivered risk scores that separated overall-survival curves 4-fold, demonstrating the potential of combining the strengths of transformers with radiomics.^32^

Complementing these large-scale studies, Zhang et al^33^ showed that an attention-augmented ConvNeXt (a next generation CNN) model achieves an AUC of 0.97 for CDKN2A/B deletion on a cohort of just 234 IDH-mutant astrocytomas, highlighting that transformer-style self-attention can still excel when data are relatively sparse. Together, these investigations demonstrate how utilizing the capacity for attention-based models to learn from an entire image, boosted by multimodal inputs of clinical data and/or fine-grain radiomic pipelines, can feasibly produce highly accurate and generalizable models to predict the molecular profiles of gliomas and close the loop between tumor biology, imaging phenotype, and actionable treatment decision.

Byeon et al^34^ extended this idea with GlioMT, an interpretable multimodal transformer that fuses MRI and basic clinical data via a frozen BERT encoder (a pre-trained language model). This BERT encoder converts clinical text into sentences, encoded into fixed embeddings, and then fused with the output of a ViT. Unlike the previously described CrossFormer and ConvNeXt models, GlioMT utilized a roll-out attention mechanism, which provides a map of the attention each token receives across the layers and thereby allowing human interpretation of which features contributed to the prediction. Trained on 1053 patients and validated on 677 more, it retained AUCs of 0.915-0.981 for IDH, 0.806-0.854 for 1p/19q, and 0.862-0.960 for WHO-grade prediction while supplying anatomically plausible attention maps that mirror radiological hallmarks such as the T2-FLAIR-mismatch sign.

However, classical ML or neural network pipelines remain valuable for narrower yet radiobiologically important endpoints where patient numbers are inherently limited. A feed-forward neural network trained on only 27 patients with paired FMISO-PET and MRI sequences successfully classified hypoxic voxels with 83% accuracy using MRI alone, while a recent radiomic Random-Forest model trained on just 86 post-stereotactic radiosurgery brain-metastasis patients now distinguishes radiation necrosis from true tumor progression with an AUC of 0.91, offering oncologists an evidence-based alternative to high-risk biopsy when deciding the next stage of treatment.^35^^,^^36^ Such lightweight models demand minimal computational resources, offer straightforward feature attribution, and can be trained on single-center datasets, making them an indispensable complement to transformer frameworks for personalized radiotherapy when the target signal is rare or the cohort small.

Despite these encouraging performance metrics, no imaging-based virtual-biopsy model for glioma has been established into routine clinical practice to date. A central barrier to translation is explainability and physician trust. Clinicians must be confident that predictions are driven by biologically and anatomically plausible tumor features rather than scanner-specific artifacts, side effects or shortcut learning, particularly when outputs may influence decisions that would otherwise rely on tissue biopsy. Under ESMO’s Basic Requirements for AI-based biomarkers in Oncology, these tools are best regarded as indirect biomarkers rather than replacements for gold-standard molecular assays.^37^ Clinical translation will therefore require stronger analytical validation against reference molecular testing, larger and more representative multicenter datasets, robust external and prospective evaluation, and reporting within recognized frameworks such as CONSORT-AI, SPIRIT-AI, DECIDE-AI, TRIPOD+AI, CLAIM, and TEHAI.^38^

### Automated staging

Automated cancer staging is a potentially highly consequential application for DL methods within the realm of radiation oncology, as TNM (tumor, node, metastasis) stage drives downstream clinical decisions—indication to treat, prognosis, dose prescription and elective nodal coverage to name a few. It could be argued that, fundamentally, automated staging requires differentiating normal tissue from tumor in the context of their anatomical position and relationships with surrounding organs (such as invasion). Therefore, automated staging may also represent a key steppingstone in the journey toward ML-driven clinical target volume (CTV) prediction and could be combined with dedicated text-based classifiers of pathology reports (such as Kefeli et al’s^39^ BB-TEN) to produce full site-specific staging. Representative studies across T-, N-, and M-staging are summarized in Table 2.

**Table 2:** Summary of representative studies for ML/DL approaches to predicting cancer staging, across T-, N-, and M- staging.

Study	Clinical context	Population	Training data size	Dataset type	Model architecture	Performance metrics
**Trabelsi et al.^40^**	Automated lung tumor segmentation and T classification using 18FDG PET/CT.	355 Patients total; 305 with T-stage labels	305 CT scans split into 243 train, 31 validation, 31 test; after augmentation, training set expanded to 1215 scans.	Lung-PET-CT-Dx: DICOM CT + 18FDG PET/CT fusion; XML bounding-box annotations.	Segmentation: modified ResNet-50. Classification: modified CNN ResNet-50 using segmented tumor regions.	Segmentation: loss test 0.009; accuracy test >0.99. Classification (global): accuracy 0.96; weighted precision 0.89; weighted recall 0.87; weighted F1 0.88.
**Fan et al.^41^**	Lung cancer T-stage (T1-T4) classification from CT; Swin-Transformer vs. CNN baselines.	Single-center cohort: 176 lung cancer patients with postoperative pathology (2021-23).	123 Training, 18 validation, 35 test	Single-center pre-operative chest CT; augmentation (flips/rotation/translation).	Compared ResNet-50, VGG16, MobileNetV2, SwinT. Transfer learning with pretrained weights; SGD; CrossEntropy.	T-stage classification: Accuracy 0.72, precision 0.76, recall 0.66, F1 0.70, AUC 0.87.
**Yang et al.^42^**	Nasopharyngeal carcinoma (NPC) gross tumor segmentation plus T-stage identification for radiotherapy planning	320 NPC patients and independent dataset of 150 patients	320 For 3-fold cross-validation; 150 independent external test	Contrast-enhanced T1-weighted MRI (CE-T1WI)	Modified 3D U-Net segmentation branch; 3D Bottleneck Transformer T-stage predictor; feature-fusion-aware module; classifier	T-stage prediction, independent dataset: accuracy 0.70, sensitivity 0.62, specificity 0.89, AUC 0.85.
**Kann et al.^43^**	Pretreatment CT-based prediction of nodal metastasis (NM) and extranodal extension (ENE) in head and neck cancer	270 Patients; 653 pathologically correlated lymph nodes	2875 CT-segmented lymph node samples for training; 124 for validation; blinded test set 131 nodes for NM and 98 nodes ≥1 cm for ENE	Pre-treatment IV contrast-enhanced neck CT with node-level pathology correlation	3D CNN; BoxNet + SmallNet combined into DualNet; compared with radiomic random forest and benchmark logistic model	NM: accuracy 85.5%, sensitivity 0.84, specificity 0.87, AUC 0.91, PPV 0.88, NPV 0.82 ENE: accuracy 85.7%, sensitivity 0.88, specificity 0.85, AUC 0.91, PPV 0.66, NPV 0.95.
**Xu et al.^44^**	Automated detection/segmentation of metastatic prostate lesions on whole-body PSMA PET/CT.	525 Whole-body [18F]DCFPyL PSMA PET/CT images	418 Training, 30 validation, 77 test	Whole-body PSMA PET and paired CT	U-Net-based CNNs (ResNet-34/VGG-16/DenseNet-121 backbones; MA-Net; UNet++). Proposed wDice loss to up-weight small lesions	wDice vs. baseline: +0.07 detection rate, +0.03 lesion-wise DSC, +0.04 lesion-wise sensitivity (all *P = .*01). Across splits mean: detection rate 0.80; 0.93 for lesions SUVmax > 5.0; median lesion-wise DSC 0.51 and 0.60 for SUVmax > 5.0

Abbreviations: 18FDG, fluorodeoxyglucose; CE-T1WI, contrast-enhanced T1-weighted imaging; NPV, negative predictive value; PET, positron emission tomography; PPV, positive predictive value; PSMA, prostate-specific membrane antigen; SGD, stochastic gradient descent; SUVmax, maximum standardized uptake value; wDice, weighted batch-wise Dice loss.

Trabelsi et al^40^ utilized a modified ResNet‑50 that performs 3D tumor segmentation on the PET/CT volume. In the last 4 layers, the authors swapped the usual lower-resolution filters for high‑resolution convolutions and added up-sampling steps that re‑expand the image before the tumor mask is drawn. The resulting voxel mask is then fed into a second ResNet‑50 that assigns the T‑category. Trained on 355 retrospective cases, with augmentation expanding the training set to 1,215 images, the model achieved a mean accuracy of 0.97 and mean per-class F1 of 0.88 across Tis-T4 disease.

Because staging depends on long-range anatomical relationships, transformer-based models are a logical development. Fan et al^41^ used a lightweight swin transformer for lung T staging, employing shifted local attention windows to exchange information efficiently across the image. The model achieved a macro-averaged F1 score of 0.70 on the held-out test set and outperformed ResNet-34, VGG-16, and MobileNet-V2 baselines trained under identical conditions.

The synergy of CNN feature locality and transformer global awareness makes their integration a promising avenue for staging, where precise margins and whole‑image context are equally critical. Yang et al^42^ introduced a 3D multi‑task network which starts with a U‑Net to perform gross tumor volume (GTV) segmentation, with the resulting tumor mask multiplied with the MRI volume. Then, a Bottleneck ViT—a ResNet‑50 whose last 3 bottleneck blocks, that is, the final 3 convolutional layers, are replaced by 3D multi‑head self‑attention layers—predicts T‑stage. A feature‑fusion‑aware attention module blends segmentation features with transformer features before classification, allowing the 2 tasks to reinforce each other. Trained on 320 contrast-enhanced T1 MRIs and externally validated on 150 additional patients, the system delivered a median dice similarity coefficient (DSC) of 0.74 for contouring and a ROC‑AUC 0.85 for staging, with 76 % accuracy for early (T1-2) and 64 % for advanced (T3-4) disease, outperforming single‑task 3D ResNet, RAN, DenseNet, and ResNeXt baselines.

Automated N-staging faces a particular ground-truth problem because nodal labels must be pathologically verified. Kann et al^43^ addressed this by training on 2875 CT-segmented lymph nodes with matched pathology in head and neck squamous cell carcinoma. Their DualNet CNN achieved an AUC of 0.91 for nodal metastasis, similar to a handcrafted-feature random forest, and improved extranodal-extension detection relative to the radiomic model.

Automated M-staging is arguably more demanding still. Unlike T- and N-staging, it may require whole body or multi-region search, detection of multiple lesions, and discrimination of small, heterogenous, low-contrast deposits from benign mimics. Reflecting this complexity, examples remain comparatively sparse. Xu et al^44^ demonstrated the value of multimodal fusion in metastatic prostate cancer using co-registered PSMA PET/CT and a loss function that up-weighted small lesions, achieving an overall lesion detection rates of 0.80 and 0.93 for lesions with maximum standardized uptake values (SUVs) greater than 5.

In sum, recent work shows the potential role of ML across every arm of TNM staging, each demonstrating shared and unique challenges. A unified end-to-end staging model will therefore likely need to weave together several strengths: multimodal fusion including functional imaging, finely detailed boundary tracing that still preserves wider context and adaptability to the nuances of tumor site. Ultimately, however, many staging pipelines still rely on a more basic prerequisite: accurate identification and delineation of tumor tissue. This makes tumor tissue segmentation the next logical component in the pathway toward more comprehensive ML-assisted radiotherapy planning.

### Tumor tissue segmentation

DL auto-segmentation of normal tissue structures has already seen extensive adoption and has shifted to guideline-endorsed clinical practice, with a joint ESTRO-AAPM guideline published in 2024 harmonizing recommendations for implementation across European and North-American centers.^16^^,^^38^ This success has garnered interest in attempts to develop techniques for automated segmentation of GTVs, as a route to acquiring the maximum value from the potential of ML technologies. Reliable delineation of tumor tissue could serve as both an essential input in radiotherapy planning (automated CTV prediction, adaptive radiotherapy) and for high throughput labeling for other ML algorithms.

However, although many lessons can and have been learnt from the journey toward improved automated normal tissue segmentation, tumor tissue presents a number of its own unique challenges. Standard U-Nets, which perform excellently for relatively stable and well imaged normal organs and tissue, can struggle with the high heterogeneity of tumor size, shape and appearance, and there can be inherent class-imbalance issues with available imaging data as voxels containing tumor usually represent a very small fraction of the overall imaged tissue. In addition, the diffuse and infiltrative patterns that tumors can display, and the need for high quality nigh-pixel wise annotation can make model training both challenging and laborious.^45^^,^^46^

Ensemble strategies may help address these challenges. The winners of the Multimodal Brain Tumor Segmentation (BrATS) Adult Glioma Challenge 2023, Ferreira et al^47^ tackled data scarcity and class imbalance by using a FTL technique of data augmentation via 3D Glioma Generative Adversarial Network (GliGAN), producing 23 049 synthetic tumor volumes from the original supplied 1470 for the challenge. In brief, GliGAN pits a Swin-UNETR (which produces a synthetic tumor volume within a real healthy MRI) against a CNN (which determines whether it’s a real or synthetic image), until the generator can mostly fool the discriminator. Swin-UNETR uses the shifted windows technique described previously to allow for inclusion of global context whilst remaining computationally efficient, but for the task of producing a voxel mask instead of classification. They used this ballooned data to train a segmentation model consisting of 3 networks implemented inside the nnU-Net framework: a standard 3D nnU-Net, a Swin-UNETR transformer (capturing long-range context), and an enlarged nnU-Net variant that had won BraTS-2021. Despite the newly introduced penalty system for false positives, this model reached DSC of 0.90, 0.87, and 0.85 for whole tumor, tumor core and enhancing tumor volumes, respectively.^48^

Given the challenges of analyzing tumor tissue and the time-intensive nature of manual annotation, the field has also looked toward foundation models (FMs). These are large neural networks trained once on diverse datasets and then fine-tuned for a range of specific tasks, a strategy proven successful in natural language processing with LLMs. In a groundbreaking article, Jun Ma et al. adapted this approach to medical image segmentation by fine-tuning the Segment Anything Model (SAM) on 1.57 million image-mask pairs across 10 imaging modalities and over 30 cancer types. The result, MedSAM, is a prompt-driven segmentation model capable of adapting to a wide range of tumor types with minimal user input. Crucially, the representational learning is completed during pre-training, allowing downstream tasks to rely on lightweight prompts such as bounding boxes or points, and thus significantly reducing manual annotation demands. For nasopharyngeal cancer segmentation, MedSAM achieved a median DSC of 0.88, out-performing standard SAM by 52% and specialist nnU-Net/DeepLabV3+ models by 15 and 23%, respectively.

Recent work has already moved beyond the first-generation MedSAM to MedSAM-2, which replaces SAM’s plain ViT backbone with the video-centric SAM-2 architecture and adds a “one-prompt segmentation” strategy for 3D studies.^49^ In a 2025 lung-CT benchmark covering >360 tumors, the bounding-box version of MedSAM-2 lifted mean tumor DSC from 0.64 with MedSAM to 0.91, and outperformed all nnU-Net and transformer baselines on the same data.^50^ However, parallel evidence warns that FMs cannot necessarily be deployed out of the box in clinical settings. For example, Nguyen et al. evaluated off-the-shelf MedSAM and its streamlined LiteMedSAM on 589 pelvic-MRI cases for normal anatomy segmentation and found they achieved mean DSC as low as 0.25 on complex structures, with little benefit from prompt engineering; only task-specific fine-tuning of LiteMedSAM raised performance to clinical parity with a bespoke nnU-Net (eg, obturator-internus DSC 0.86 vs. 0.25).^38^^,^  ^51–53^

### CTV prediction

The CTV encompasses the visible tumor (GTV) and adjacent tissue judged at risk of microscopic disease.^54^ CTV prediction therefore combines many of the tasks discussed above: tumor delineation, assessment of local invasion, nodal involvement, and normal anatomy, alongside disease-site guidelines and patient-specific context.^55^^,^^56^ In other words, it is not simply an image-segmentation problem but a clinically contextualized decision-making task.

The recent LLMSeg system proposed by Oh et al^57^ represents a leap in context-aware, multimodal CTV prediction. LLMSeg, trained to perform breast cancer CTV delineation on ≈700 breast cancer cases across 3 institutions, conditions a 3D residual U-Net on a frozen LLM (Llama-7B) via bidirectional cross-attention, allowing the network to ingest both structured clinical text (tumor stage, surgery, laterality, guideline snippets) alongside CT images. Crucially, this promptable LLMSeg demonstrated a number of marked advantages over Vision-Only Models (VOMs). First, it maintained a DSC above 0.82 across both external test sets, while the VOMs dropped to 0.44 with the external set including a different acquisition modality to which it was trained. Second, it demonstrated remarkable training efficiency, maintaining DSC just under 0.8 even after the training set was pruned to 20% of its original size (while VOMs dropped to below 0.4). Third, LLMSeg vastly outperformed VOMs in terms of blinded expert evaluation, particularly for laterality and volume definition. Finally, ablation experiments showed that changing just the textual prompt (eg, up-staging N-class) caused the model to add or remove elective nodal volumes in the expected fashion.

These approaches consider target volume contouring as a single hard classification task, rather than more akin to a probability distribution problem. Bridging this gap may both introduce elements more in keeping with the approach of a radiation oncologist, and also aid in the confidence in, and adoption of automated target volume segmentation models by highlighting inevitable uncertainties during model inference. Several methods have been considered to produce “probability maps,” including probabilistic U-Nets and Bayesian approximation using Monte Carlo drop-out.^58–61^

Looking ahead, CTV delineation is likely to be delegated not to a single network but to an agentic, tool-using workflow that can reason over clinical context, choose the right model or selection of models, integrate modalities as required, coordinate downstream checks, and highlight regions of uncertainty to the treating radiation oncologist (Figure 2).^62^

**Figure 2:**
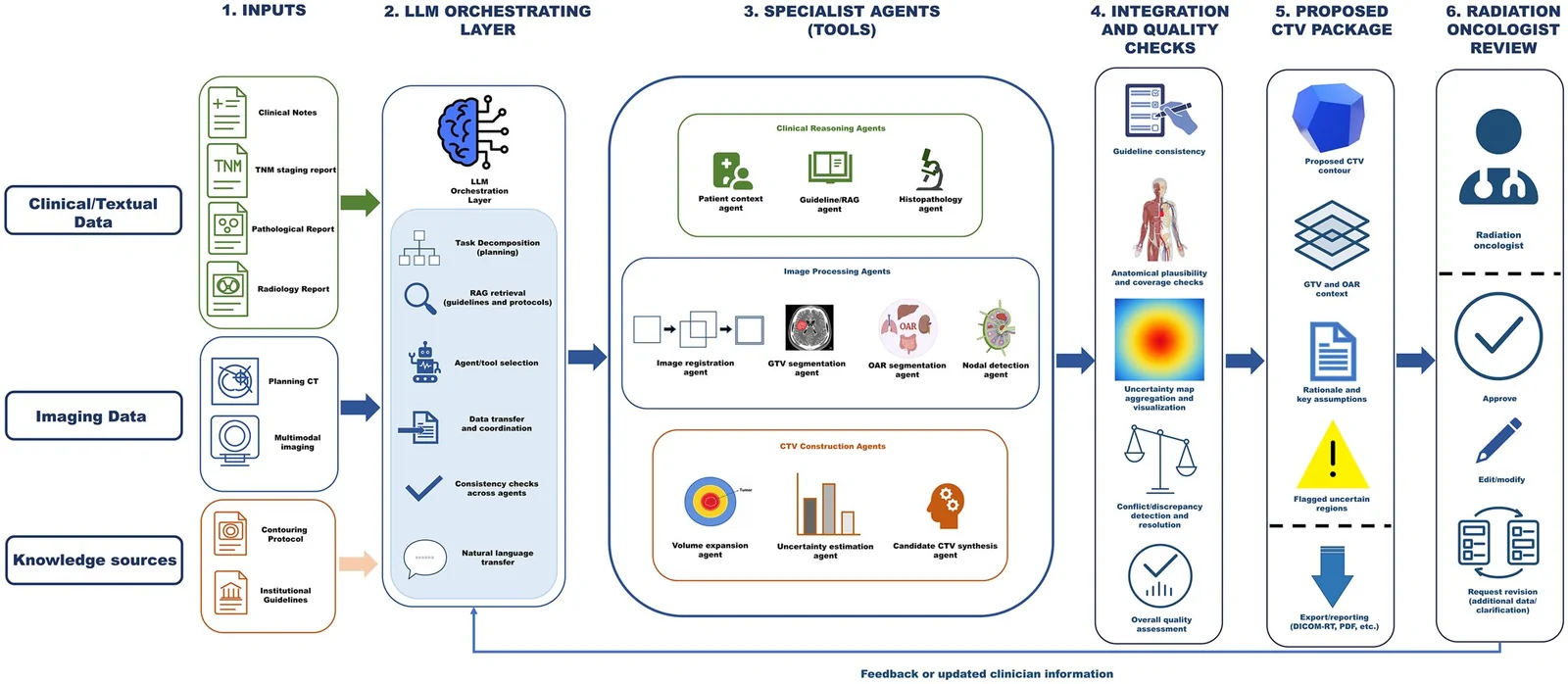
Proposed agentic workflow for context-aware CTV delineation. Abbreviations: CTV, clinical target volume; DICOM-RT, digital imaging and communications in medicine for radiation therapy; GTV, gross tumor volume; LLM, large language model; RAG, retrieval-augmented generation.

This differs from a standard multimodal segmentation model. A multimodal model fuses predefined inputs, such as CT, MRI, PET, and structured text, within a fixed trained network. An agentic workflow instead adds an orchestration layer that decomposes the clinical task, retrieves site-specific guidance, selects specialist tools, transfers outputs between them, and applies checks before clinician review.

This direction is supported by the evolution of LLMs from sequence generators into systems capable of reasoning, planning, and tool use.^63^ In CTV delineation, an LLM could therefore function as a supervisory layer that coordinates specialized components within a contouring workflow.^64^ A Retrieval-Augmented Generation (RAG) framework may be particularly useful as part of this workflow allowing an LLM to ground its outputs in retrieved protocols, guidelines, and clinical documents rather than pretrained knowledge alone.^65^

Operationally, this supervisory layer would not replace the segmentation network. It would sit above specialized models and services: first calling on specialized LLMs to parse text-based patient information (eg, clinic letter, MDT documentation, pathology/radiology reports) and retrieving site-specific contouring guidance through RAG; then calling dedicated tools for OAR segmentation, GTV segmentation, nodal detection, image registration, and margin expansion; then comparing outputs against guideline constraints and uncertainty maps before returning a proposed CTV and a set of flagged regions for radiation oncologist review. Such a framework would allow each specialized unit to utilize the relative advantages of algorithmic developments in their domain, while CTV prediction is viewed as less a 1-step segmentation task than a structured reasoning problem with a segmentation endpoint.

### Applications beyond the oncologist role

Until now we have centered on image- and patient-derived information that supports the radiation oncologist’s diagnostic and contouring tasks. The same data landscape, however, is beginning to power physics-driven tasks traditionally handled by dosimetrists and medical physicists, including rapid dose prediction and adaptive planning. By introducing knowledge of the treatment beam, researchers can create synthetic, Hounsfield-unit-accurate CT volumes on which fast dose calculations run. One stand-out example is the use of cycle-consistent generative adversarial networks to convert daily cone-beam CTs into dosimetrically faithful synthesized CTs, as demonstrated by Deng et al.^66^ However, comprehensive analysis of these veins of research, including physics-informed models, would warrant a manuscript in their own right, yet they represent essential components to developing truly end-to-end workflow acceleration in radiation oncology.

## Conclusion

The diverse use cases discussed—from virtual biopsies and automated staging through normal and tumor segmentation—can be viewed as successive way points on a single roadmap: building an increasingly rich, multi-scale map of medical image space. Each methodological innovation enlarges that map’s vocabulary (radiomics), grammar (CNNs and transformers), and capacity for context (multimodal, LLM-conditioned models such as LLMSeg). Together, they bring us closer to the long-sought goal of robust, generalizable CTV prediction ie, aware of anatomy, tumor biology, and clinical context.

Across this review, we have traced how ML in radiation oncology has stretched beyond “plain” U-Nets and into a spectrum of task-specific and multimodal architectures. Importantly, each leap in sophistication adds to, rather than replaces, the tools that came before, suggesting that future workflows will blend specialized networks for individual tasks with broader foundational models or agentic frameworks. While many of these technologies are innovative and hold great potential, implementation of these tools beyond normal tissue auto-segmentation into clinical practice remains very limited. Ongoing efforts should utilize the developing international frameworks and guidance for development, assessment, and validation of ML algorithms as they apply to radiation oncology.
